# MFSD12 affects glycosphingolipid metabolism by modulating lysosome homeostasis

**DOI:** 10.1093/procel/pwac034

**Published:** 2022-08-11

**Authors:** Yun Hong, Zheng Tian, Liangjie Jia, Yiguo Wang

**Affiliations:** MOE Key Laboratory of Bioinformatics, Tsinghua-Peking Center for Life Sciences, School of Life Sciences, Tsinghua University, Beijing 100084, China; MOE Key Laboratory of Bioinformatics, Tsinghua-Peking Center for Life Sciences, School of Life Sciences, Tsinghua University, Beijing 100084, China; MOE Key Laboratory of Bioinformatics, Tsinghua-Peking Center for Life Sciences, School of Life Sciences, Tsinghua University, Beijing 100084, China; MOE Key Laboratory of Bioinformatics, Tsinghua-Peking Center for Life Sciences, School of Life Sciences, Tsinghua University, Beijing 100084, China

**Dear Editor,**

Glycosphingolipid (GSL) catabolism is strictly regulated in a sequential manner by lysosomal hydrolases with the help of lipid-binding proteins. Impairments of the catabolic pathway cause the onset of lysosomal storage diseases (LSDs), which are characterized by the aberrant accumulation of GSLs (Hannun and Obeid 2018; Breiden and Sandhoff 2019). The primary LSDs occur through the well-known mechanism of inherited defects in genes encoding the catabolic enzymes (or their related cofactors) which reside in the lumen of lysosomes (Hannun and Obeid 2018; Breiden and Sandhoff 2019). However, in some LSDs, the inherited mutations affect other proteins, such as those residing in the lysosomal membrane; these mutations are associated with defects in trafficking and fusion in the endocytic system (Platt et al. 2012). However, the non-lumenal lysosomal membrane proteins involved in GSL metabolism remain to be identified.

The lysosome is a central organelle for catabolic function in energy metabolism, nutrient sensing, and nutrient recycling in response to nutritional stress, and homeostatic mechanisms are critical for maintaining the catabolic function of lysosomes (Mizushima and Komatsu 2011; Settembre et al. 2013). The internal environment of the lysosome is controlled by lysosomal membrane proteins, such as vacuolar-type H^+^-ATPase (V-ATPase), which actively pump hydrogen ions into the lysosome to maintain the highly acidic environment within the lysosomal lumen for normal catabolism (Mindell 2012). mTOR, a nutrient and energy sensor, regulates lysosomal function via transcription factor EB (TFEB)-mediated gene expression (Settembre et al. 2013). TFEB, a downstream target of mTOR and one of microphthalmia family of transcription factors, shuttles between the nucleus and cytoplasm to control the expression of genes required for lysosome biogenesis and autophagy (Settembre et al. 2013). Although many genes are known to play critical roles in lysosomal homeostasis, it is still unclear whether they affect GSL metabolism.

To identify which lysosomal transporters are involved in GSL metabolism, we tested the effect of 82 lysosomal membrane-localized transporters on expression of β-galactosidase (GLB1), an enzyme in GSL catabolism (Hannun and Obeid 2018; Breiden and Sandhoff 2019) (Fig. S1A). We established stable expression of the reporter *GLB1*-Luc in the human intestinal epithelial cell line HIEC-6. *GLB1*-Luc consists of a luciferase reporter gene linked to the promoter of the β-galactosidase gene *GLB1*. The cells were transfected with a siRNA library targeting 82 human lysosomal transporter-encoding genes and a control vector, RSV-Luc, to monitor transfection efficiency and cell viability (Fig. S1B). We performed this screen twice with very good reproducibility (Spearman *R* = 0.98) and identified 12 genes that increased *GLB1c-*Luc activity by more than 2-fold (Fig. S1C and Table S1). Surprisingly, knockdown of *MFSD12*, a gene involved in pigmentation and cysteine transport (Crawford et al. 2017; Adelmann et al. 2020), increased *GLB1-*Luc activity almost 3-fold (Fig. S1C and Table S1). These results suggest that MFSD12 may modulate GSL metabolism via regulation of GLB.

To explore the possible roles of MFSD12 in GSL metabolism, we tested the expression of MFSD12 in different adult mouse tissues by quantitative PCR (qPCR) and immunoblot (Fig. S1D and S1E). MFSD12 was highly expressed in the intestine and skin (Fig. S1D and S1E), indicating that MFSD12 may have an important function in these tissues. Immunostaining results showed that MFSD12 was co-localized with the lysosomal integral membrane protein LAMP1 (Fig. S1F), which is consistent with the previous report (Crawford et al. 2017; Adelmann et al. 2020). To further confirm the localization of MFSD12 in tissues, we generated MFSD12-GFP knock-in mice (Fig. S1G). Similar to the cellular localization, MFSD12 was localized at the lysosomal membrane in the intestine (Fig. S1H).

To investigate the transmembrane orientation of MFSD12, we made an MFSD12 construct with a FLAG-tag at its N-terminus and an HA-tag at its C-terminus. Lysosomes were purified from HEK293T cells with FLAG-MFSD12-HA expression, and the fluorescence signals in unpermeabilized and Triton X-100-permeabilized lysosomes were detected by flow cytometry. As shown in Fig. S1I, both the FLAG and HA signals were detected in unpermeabilized and permeabilized lysosomes, indicating that both the N-terminus and C-terminus of MFSD12 are exposed to the cytoplasmic side. As a positive control, we checked the orientation of LAMP1 by the same assay. Consistent with the previous report (Fukuda 1991), the C-terminus of LAMP1 faces the cytoplasm and its N-terminus faces the lysosome lumen (Fig. S1J). Taken together, these results suggest that lysosome-localized MFSD12 may affect GSL metabolism.

*MFSD12* was shown to be involved in pigmentation (Crawford et al. 2017), which is related to its high expression in skin. However, the physiological roles of MFSD12 in other tissues are unknown. Since MFSD12 is highly expressed in the intestine, we generated mice with intestine-specific knockout (CKO) of *Mfsd12* (*Mfsd12*^fl/fl^; *Vil1*-Cre^+^) to investigate the function of *Mfsd12* in the intestine (Fig. 1A–D). Both CKO male mice and female mice showed partial embryonic lethality after embryonic day 14.5 (E14.5) and around 20% of CKO mice survived to adulthood (Fig. S2A). The onset of embryonic lethality occurred between the E14.5 and E18.5 stages (Fig. S2A), which indicates that MFSD12 may be required for organ formation. After birth, the CKO mice survived beyond 18 months without significant spontaneous death. Together, these results show that MFSD12 is required for embryonic development.

**Figure 1.**
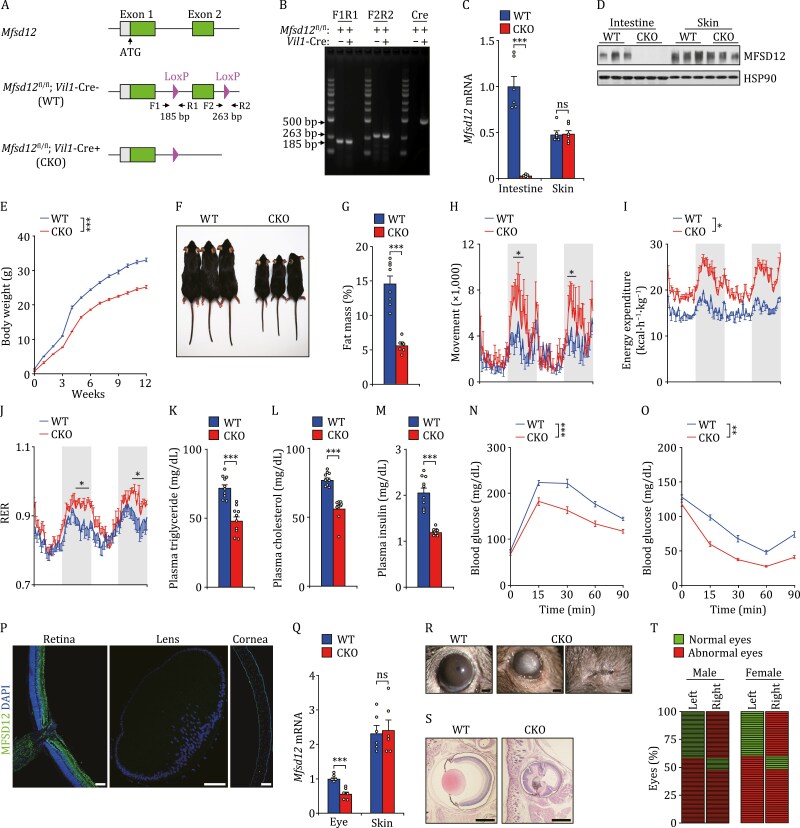
*Mfsd12* deficiency impairs mouse metabolism and disrupts eye function. (A) Generation of *Mfsd12* CKO mice. The positions of genotyping primers are shown. (B) PCR analysis showing *Mfsd12* fragments generated from WT and *Mfsd12* CKO mice. (C–D) qPCR results (C) and immunoblots (D) showing knockout of *Mfsd12* in the intestine of CKO mice. (E–F) Body weight curves (E) and photographs (F) of WT and CKO male mice. (G–M) Effect of *Mfsd12* CKO on fat mass ratio (G), movement (H), energy expenditure (I), RER (J), plasma triglyceride levels (K), plasma cholesterol levels (L), and plasma insulin levels (M). (N–O) Results of glucose tolerance test (N) and insulin tolerance test (O) from WT and *Mfsd12* CKO mice. (P) Immunostaining showing the expression of MFSD12 in the retina, lens, and cornea of eyes from adult MFSD12-GFP knock-in mice. Scale bars, 100 μm. (Q) qPCR results showing relative mRNA levels of *Mfsd12* in eye extracts from adult WT and CKO mice. (R–T) Effect of *Mfsd12* deficiency on the eyes from adult mice. Gross morphology (R), hematoxylin and eosin sections (S), and the percentage of normal/abnormal eyes (T) are shown. Each line in (T) shows the status of the left and right eye from one mouse. Scale bars, 1 mm. Data are shown as mean ± SEM. Comparison of different groups was carried out using Student’s *t*-test (C, G, K–M, Q) or two-way ANOVA (E, H–J, N–O). *P < 0.05, **P < 0.01, ***P < 0.001. ns, no statistical significance. *n* = 6 male mice (C, Q), *n* = 8 male mice (E, G–O), *n* = 86 male mice and *n* = 60 female mice (T).

Compared to WT mice, *Mfsd12* CKO mice had reduced body weight, smaller body size, and lower fat mass (Figs. 1E–G and S2B). In addition, *Mfsd12* deficiency enhanced mouse movement, energy expenditure, and respiratory exchange ratio (RER), but had no effect on food intake, water intake, and intestine morphology (Figs. 1H–J and S2C–E). Reduced plasma triglyceride levels, cholesterol levels, and insulin levels were observed in *Mfsd12* CKO mice (Fig. 1K–M), which is suggestive of enhanced insulin sensitivity in these animals. Consistent with this observation, *Mfsd12* deficiency improved glucose tolerance and insulin tolerance (Fig. 1N and 1O). Together, these results indicate that MFSD12 is critical for metabolism.

Since *Vil1* was previously reported to be detected in the eyes as well as the intestine (De Velasco et al. 1999), we tested the expression of MFSD12 in the eyes by using MFSD12-GFP knock-in mice. As shown in Fig. 1P, MFSD12 was expressed at a high level in the retina, and at a relatively low level in lens and cornea. In addition, the expression of *Mfsd12* in the eyes was dramatically reduced in *Mfsd12* CKO mice (Fig. 1Q). Strikingly, some *Mfsd12* CKO mice from E14.5 showed complete absence of eyes, extremely reduced eye size or a malformed pupil (Fig. S3A and S3B). No *Mfsd12* CKO mouse embryos had two healthy eyes, but ~30% *Mfsd12* CKO embryos had one normal eye (Fig. S3C). Adult *Mfsd12* CKO mice displayed microphthalmia, lens–corneal adhesion and total lens opacity, and had at least one malformed eye (Fig. 1R–T). Together, these results indicate that MFSD12 is necessary for eye development and function.

Since MFSD12 may regulate GSL metabolism, we employed thin-layer chromatography (TLC) and liquid chromatography–mass spectrometry (LC-MS) approaches to determine the intestinal profiles of three GSL derivatives, gangliosides GM1–3. Whole-cell, cytosolic, and lysosomal fractions were prepared from intestinal cells from WT and CKO mice. The fractions were separated by TLC and the ganglioside content was analyzed by LC-MS (Fig. 2A–D). The lysosomal fractions of WT and CKO mice yielded three prominent TLC bands (from top to bottom, Band 1 to Band 3) which were identified by LC-MS as GM3, GM2 and GM1 respectively (Fig. 2A–D). Compared to WT mice, *Mfsd12* CKO mice had increased levels of both GM2 and GM3 but not GM1 in the lysosomes, whereas ganglioside species in whole-cell lysates were similar (Fig. 2A and 2E). These results indicate that MFSD12 modulates ganglioside catabolism and *Mfsd12* deficiency results in the aberrant accumulation of GM2 and GM3 in the lysosomes.

**Figure 2.**
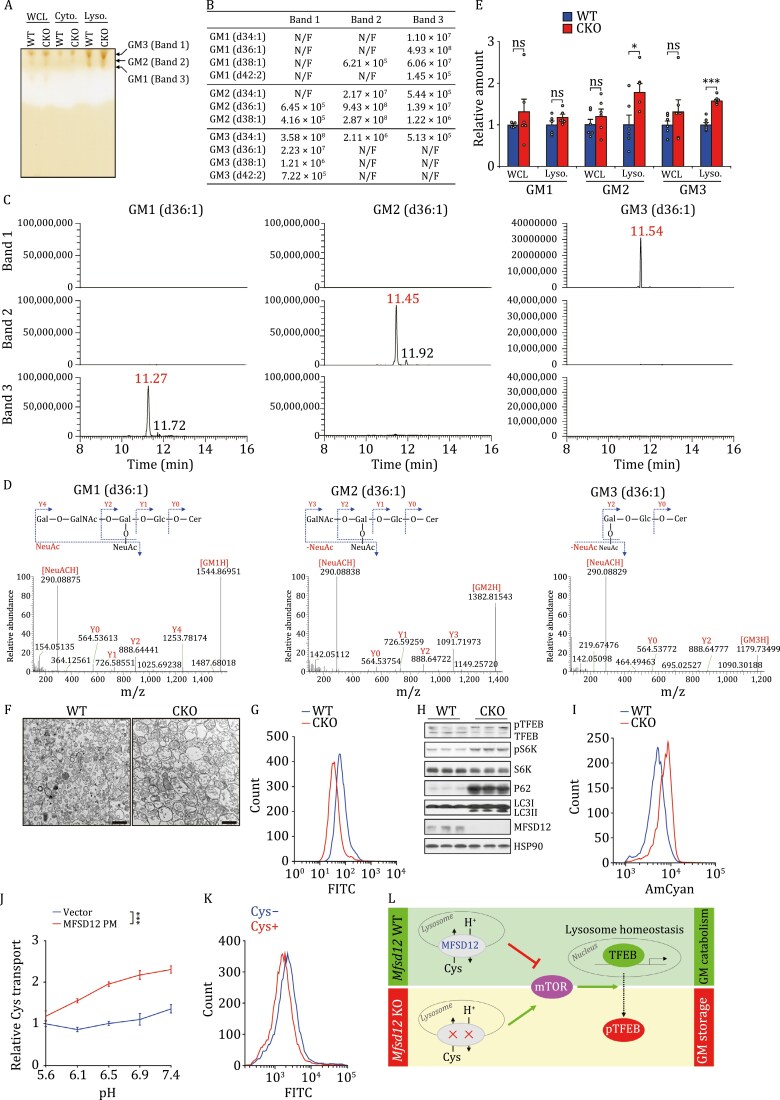
Enhanced accumulation of gangliosides and attenuated lysosomal function in *Mfsd12*-deficient mice. (A) TLC results showing the relative amounts of gangliosides GM1–3 in intestinal lysosomes from WT and CKO mice. WCL, whole-cell lysate; Cyto., cytosolic fraction. Lyso., lysosome. (B) The identified ganglioside species and the relative amounts of ganglioside species corresponding to the labeled bands (A) are shown. (C–D) Representative chromatograms (C) and high-resolution MS spectra (D) showing the identified ganglioside species. (E) Relative contents of GM1, GM2, and GM3 in the intestinal WCL and lysosome fractions from WT and CKO mouse detected by liquid chromatography–mass spectrometry: *n* = 6 mice. (F) Representative electron microscopy (EM) images showing the effect of *Mfsd12* deficiency on lysosome phenotypes of mouse intestine. Scale bars, 1 μm. (G) Effect of *Mfsd12* deficiency on lysosomal function. (H) Immunoblots showing the effect of *Mfsd12* deficiency on autophagy markers and TFEB signaling in the intestine from WT and CKO mice. (I) Effect of *Mfsd12* deficiency on the pH of intestinal lysosomes from WT and CKO mice. (J) Effect of pH on cysteine transport by MFSD12 PM in HEK293T cells. MFSD12 PM (L253A/L254A) is a plasma membrane-localized mutant of MFSD12. *n* = 3. (K) Effect of cysteine transport on cellular pH in HEK293T cells. (L) MFSD12 transports cysteine into the lysosomes with H^+^ export from the lysosomes. This maintains lysosome homeostasis via the mTOR–TFEB-mediated pathway and thereby modulates the catabolism of glycosphingolipids. *Mfsd12* deficiency abolishes cysteine import into the lysosomes and attenuates H^+^ export from the lysosomes. This affects TFEB-mediated lysosome homeostasis by activating mTOR and inactivating TFEB, and results in accumulation of gangliosides in the lysosomes. Data are shown as mean ± SEM. Comparison of different groups was carried out by using Student’s *t*-test (E) or two-way ANOVA (J). *P < 0.05, ***P < 0.001. ns, no statistical significance.

MFSD12 was recently reported as a cysteine transporter that imports cysteine from the cytoplasm into the lysosomes (Adelmann et al. 2020). Consistent with this conclusion, significantly reduced levels of cysteine and cystine (the oxidized dimeric form of cysteine) were detected in the lysosomes of *MFSD12* knockdown HEK293T cells (Fig. S4A–C). To investigate the underlying mechanisms by which MFSD12 affects GSL catabolism, we examined the morphology and function of intestinal lysosomes. In the intestine from *Mfsd12* CKO mice, the lysosomes were grossly swollen and contained abundant vacuolar structures (Fig. 2F), which indicates that lysosomal function is impaired. This was further confirmed by the weaker fluorescence signal in the lysosomal intracellular activity assay (Fig. 2G).

Lysosome homeostasis is critical for maintaining the catabolic function of lysosomes (Mizushima and Komatsu 2011; Settembre et al. 2013). Consistent with previous reports of LSDs (Mizushima and Komatsu 2011), autophagy flux was blocked in *Mfsd12* CKO mice, evidenced by increased levels of LC3-II (accumulation of autophagosomes) and P62 (autophagic substrate) (Fig. 2H). Interestingly, activation of mTOR, evaluated by phospho-S6K (pS6K), and enhanced phosphorylation of TFEB (pTFEB) also occurred (Fig. 2H), which indicates that lysosome homeostasis is impaired in *Mfsd12* CKO mice.

Since an acidic internal environment is critical for lysosomal function (Mindell 2012), we tested the pH of intestinal lysosomes from WT and *MFSD12* CKO mice using the lysosensor probe. As shown in Fig. 2I, *Mfsd12* CKO mice showed a stronger lysosensor probe signal, indicating that the lysosomes were more acidic. These results suggest that MFSD12 may affect lysosomal H^+^ transport. To test this hypothesis, we generated a plasma membrane-localized MFSD12 mutant (MFSD12 PM) (Adelmann et al. 2020) (Fig. S4D). The transport of cysteine by MFSD12 PM showed a positive correlation with an increased pH (Fig. 2J), indicating that MFSD12 transport cysteine in a reverse H^+^ gradient. Consistent with these results, cysteine transport increased the cellular pH value (Figs. 2K and S4E). Together, these results indicate that MFSD12 transports cysteine in an H^+^ gradient-dependent manner and then modulates lysosome homeostasis via the mTOR–TFEB pathway.

GSL catabolism in the lysosome is fulfilled by hydrolases in a step-wise fashion, and genetic defects in GSL hydrolases result in LSDs (Hannun and Obeid 2018; Breiden and Sandhoff 2019). The aberrant accumulation of GSLs by defective hydrolases caused impaired lysosomal function and organism defects, such as developmental delay, short stature, and eye malformation (Hannun and Obeid 2018; Breiden and Sandhoff 2019). Most LSDs result from mutations in genes encoding lumenal lysosomal hydrolases. However, some LSDs are also caused by mutations in genes encoding lysosomal membrane proteins. Niemann-Pick type C disease (NPC) results from deficiency of the cholesterol transporter of the lysosomal membrane cholesterol export protein NPC1, which leads to intra-lysosomal accumulation of cholesterol and GSLs (Saftig and Klumperman 2009; Platt et al. 2012). However, the other non-lumenal lysosomal membrane proteins involved in GSL metabolism remain to be identified. In this study, we identified that MFSD12 transports cysteine into lysosomes following H^+^ export from the lysosomes, which maintains lysosome homeostasis via an mTOR–TFEB-mediated pathway and thereby modulates the catabolism of GSLs (Fig. 2L). Deficiency of *Mfsd12* results in attenuated lysosomal function by disrupting cysteine-coupled H^+^ efflux from the lysosomes, which further activates mTOR and inhibits TFEB by sequestering it in the cytoplasm via phosphorylation. Thus, disruption of TFEB-mediated lysosomal function results in enhanced accumulation of GSL derivatives (gangliosides). Correspondingly, deficiency of *Mfsd12* leads to a higher lethality rate during mouse development, and surviving mice have congenital eye malformations. Of course, it is possible that the disruption of TFEB interaction with other microphthalmia family of transcription factors accounts for the congenital eye malformations. Our results demonstrate that MFSD12 as a cysteine transporter modulates lysosome homeostasis and GSL metabolism. These findings greatly expand the understanding of LSD pathogenesis and provide new insights into targeted therapy.

Previously, MFSD12 mutation was reported to affect skin color in Africans (Crawford et al. 2017). In contrast to other genes related to human pigmentation, including *TYR* and *SLC45A2* which are only expressed in the skin (Uhlen et al. 2015), *MFSD12* is widely expressed in other tissues and may function in non-skin tissues. It was reported that MFSD12 imports cysteine into melanosomes or lysosomes, which is used in melanin synthesis (Adelmann et al. 2020). We found that MFSD12 is expressed in the intestine to modulate GSL catabolism. Interestingly, melanin production is also affected by GSLs (Sprong et al. 2001). Of course, further work is necessary to address the links between GSL catabolism and cysteine transport in the skin. Although *Vil1*-Cre is usually used as a tool to knock out genes in the intestine, the target gene may also be deleted in the eye by this Cre line because *Vil1* is expressed in the eyes (De Velasco et al. 1999). Notably, the eye malformations we observed in *Mfsd12*-deficient mice correspond to the ophthalmic phenotypes described in human cases of LSDs (Hannun and Obeid 2018).

Cysteine levels regulate mTOR activity dynamically via multiple mechanisms. A recent study showed that increasing the cytosolic content of cysteine via SLC7A11 (solute carrier family 7 member 11) can promote mTOR activation (Meng et al. 2020; Zhang et al. 2021). In *MFSD12-*deficient cells, import of cysteine into the lysosomes is blocked, and the increased cytosolic cysteine level may account for mTOR activation (Fig. S4a). The acidic environment of the lysosome is mainly generated by the v-ATPase, which transports H^+^ ions into the lysosomal lumen by consuming ATP (Mindell 2012). In this study, we found that MFSD12 transports cysteine followed by H^+^ export from the lysosomes. In addition, multiple subunits of v-ATPase were identified to affect *GLB1*-Luc activity (Table S1). These results suggest that a relatively stable lysosomal pH is critical for lysosomal function. Our data suggest that MFSD12 may help to regulate lysosomal pH, which consequently affects mTOR signaling.

Taken together, our results demonstrate that MFSD12 links cysteine transport to lysosome homeostasis via the mTOR–TFEB pathway and thereby modulates GSL catabolism. Moreover, this study greatly expands our knowledge of the lysosomal membrane proteins that may be involved in the onset of LSDs. Our study also suggests the possibility of treating GSL storage diseases by modulating MFSD12 activity.

## Supplementary Material

pwac034_suppl_Supplementary_Material

pwac034_suppl_Supplementary_Table

## Footnotes

This work was supported by grants from the Ministry of Science and Technology of the People's Republic of China (2021YFA0804800), Tsinghua University Initiative Scientific Research Program (2021Z11JCQ016) and the National Natural Science Foundation of China (82088102, 31625014).

The authors have no conflicts of interest to declare that are relevant to the content of this article.

All institutional and national guidelines for the care and use of laboratory animals were followed. All mouse experiments were approved by the Animal Care and Use Committee at Tsinghua University.

All the authors give their consent for the publication of this study.

The datasets and materials generated during the current study are available from the corresponding author on reasonable request.

Y.H. measured the metabolic phenotype of Mfsd12 IKO mice. Z.T. investigated the mechanism of lysosomal impairment. L.J. analyzed the lysosomal transporters. Y.W. conceived, designed and supervised this study. Y.W., Y.H., and Z.T. wrote the paper. All authors reviewed and commented on the manuscript.
