# Correction to: MFSD12 affects glycosphingolipid metabolism by modulating lysosome homeostasis

**DOI:** 10.1093/procel/pwaf077

**Published:** 2025-09-09

This is a correction to: Yun Hong, Zheng Tian, Liangjie Jia, Yiguo Wang, MFSD12 affects glycosphingolipid metabolism by modulating lysosome homeostasis, *Protein & Cell*, Volume 14, Issue 6, June 2023, Pages 459–463, https://doi.org/10.1093/procel/pwac034

In the originally published online version of this manuscript, the published supplementary data file was errored, and lacked figures. A corrected supplementary data file is now attached to this notice.

This emendation is made within this correction notice to preserve the version of record.

