# Supplementary material for: MFSD12 affects glycosphingolipid metabolism by modulating lysosome homeostasis
Source: Protein Cell. 2022 Aug 11;14(6):459–63. doi: 10.1093/procel/pwac034 (PMC10246716; doi:10.1093/procel/pwac034)

Table S1: Results of the siRNA screen to identify genes that modulate *GLB1*-Luc in HIEC-6 cells.
Information about lysosomal transporters was extracted from the Uniprot database.

Figure S1: MFSD12 is a lysosome-localized regulator of glycosphingolipid metabolism.
(A) Schematic showing the role of GLB1 in the GSL catabolism pathway. GM1-3, gangliosides GM1-3; LacCer, lactosylceraminde; GlcCer, glucosylceramide; GalCer, galactosylceramide; GBA, glucocerebrosidase; GLB1, β-galactosidase; HEXA, β-hexosaminidase subunit α; NA, neuraminidase.
(B) siRNA library screening for lysosome-localized transporters that modulate the activity of *GLB1*-Luc in HIEC-6 cells.
(C) Correlation between the two replicates of the primary screen, using a siRNA library targeting 82 transporter-encoding genes. Results are shown as fold change (on a $\log_2$ scale) of *GLB1*-Luc activity in siRNA knockdown cells relative to cells transfected with non-targeting siRNA. Spearman's rank correlation coefficient (r = 0.98) between the two replicates is shown.
(D) qPCR results showing relative mRNA levels of *Mfsd12* in different mouse tissues. Data are shown as mean ± SEM. n = 5 mice.
(E) Immunoblot showing the expression of MFSD12 protein in different mouse tissues.
(F) Immunostaining showing cellular colocalization of MFSD12-HA and LAMP1-mCherry in Cos7 cells. Nuclei are stained with DAPI (4,6-diamidino-2-phenylindole). Scale bar, 10 μm.
(G) Generation of MFSD12-GFP knock-in mice.
(H) Immunostaining showing colocalization of MFSD12-GFP and LAMP1 in the intestinal tissue of an MFSD12-GFP knock-in mouse. Scale bar, 10 μm.
(I) Left panel: Schematic showing the topology and orientation of MFSD12 at the lysosome membrane. The middle panel and the right panel show the signals detected by flow cytometry in unpermeabilized and permeabilized lysosomes, respectively. Lysosomes were purified from HEK293T cells expressing FLAG-MFSD12-HA, then left unpermeabilized or treated with Triton X-100.
(J) Left panel: Schematic showing the topology and orientation of LAMP1 at the lysosomal membrane. The middle and right panels show the signals detected by FACSCalibur Cytometer. Lysosomes were purified from HEK293 cells expressing LAMP1-HA, and then left unpermeabilized or permeabilized with Triton X-100.

Figure S2: Characterization of *Mfsd12* knockout mice.
(A) Survival of embryonic and postnatal mice. The percentage of embryonic and postnatal *Mfsd12* WT and CKO mice at different developmental and postnatal stages.
(B) Body weight curves of WT and CKO female mice.
(C-D) Effect of *Mfsd12* CKO on food intake **(C)** and water intake **(D).**
(E) Intestinal sections from WT and CKO mice stained with haematoxylin and eosin (left) and ALPI (intestinal alkaline phosphatase, right). Scale bars, 100 μm.
Data are shown as mean ± SEM. Comparison of different groups was carried out using Student's *t*-test **(B-C)** or two-way ANOVA **(D)**. ***$P < 0.001$. *NS*, no statistical significance. n = 8 mice.

Figure S3: Effect of *Mfsd12* deficiency on eye morphology from E14.5 embryos.
(A-C) Gross morphology **(A)**, haematoxylin and eosin sections **(B)** and the percentage of normal/abnormal eyes **(C)** are shown. Each line in **(C)** shows the status of the left and right eye from one mouse. n = 29 male mice and n = 30 female mice. Scale bars, 500 μm.

Figure S4: Blunted cysteine transport into lysosomes in *MFSD12* knockdown cells.
(A-B) Effect of *MFSD12* knockdown on cysteine levels **(A)** and cystine levels **(B)** in whole-cell lysate (WCL), cytosolic fractions (Cyto.) and purified lysosomes (Lyso.) of HEK293T cells.
(C) qPCR results showing the knockdown efficiency of *MFSD12* in HEK293T cells.
(D) Cellular localization of WT MFSD12 and the PM mutant (L253A/L254A) in Cos7 cells. Scale bars, 10 μm.
(E) [$^{13}C_3$, ^{15}N]Cysteine uptake in HEK293T cells expressing MFSD12 PM.
Data are shown as mean ± SEM. Comparison of different groups was carried out using Student's *t*-test. $^*P < 0.05$, $^{**}P < 0.01$, $^{***}P < 0.001$. *NS*, no statistical significance. n = 3.

Materials and Methods
Mouse strains and experiments
Mice carrying a floxed allele of *Mfsd12* were obtained from Viewsolid Biotech. *Mfsd12$^{fl/fl}$*; *Vil1*-Cre mice were generated by crossing *Mfsd12$^{fl/fl}$* mice with *Vil1*-Cre mice. All mice used were maintained in a C57BL/6 strain background. PCR genotyping of *Mfsd12* knockout mice was performed with primers that detect the following: 5' LoxP site of the targeted *Mfsd12* allele: forward, 5'-GTATGATGGACGCTGGAG-3'; reverse, 5'-TAGGTGGCTCAGAGTCAA-3'. 3' LoxP site of the targeted *Mfsd12* allele: forward, 5'-GAATGAGTGCCAGGTGAC-3'; reverse, 5'-CTGGTTGGTGGTGGTACA-3'. Cre: forward, 5'-TGGGCGGCATGGTGCAAGTT-3'; reverse, 5'-CGGTGCTAACCAGCGTTTTC-3'. MFSD12-GFP knock-in mice were obtained from Biocytogen. PCR genotyping was performed with primers that detect the following: forward, 5'-CATTCGCAACCGTGGGTCTCCATTA-3'; reverse, 5'-TAGGCTGGTGCATCCCTGTCAAAAG-3'. Mice were housed in a temperature-controlled environment using a 12 hr light/12 hr dark cycle in a temperature-controlled environment with free access to food and water. Mice were maintained with all relevant ethical regulations for animal testing and research. All mouse experiments were approved by the Animal Care and Use Committee at Tsinghua University.
Indirect calorimetry, physical activity and food intake
Energy expenditure, respiratory exchange ratio (RER), physical activity, food intake and water intake were simultaneously measured for individually housed mice with a PhenoMaster system (TSE Systems). Mice were allowed to acclimatize in the chambers for at least 24 hrs. Food and water were provided *ad libitum* in the appropriate devices and measured by the built-in automated instruments. Relative fat mass was measured with an EchoMRI analyzer.
Metabolic studies and histology
Blood glucose values were determined using a LifeScan automatic glucometer. Glucose tolerance tests (GTT) were performed by intraperitoneal (i.p.) administration of glucose (1 g·kg^{-1}) after overnight fasting. Insulin tolerance tests were performed by i.p. injection of human regular insulin (1 U kg^{-1}) after 5-hr fasting. Plasma triglyceride levels (TR0100,

Sigma), plasma cholesterol levels (ab65390, Abcam) and plasma insulin levels (10-1247-01, Mercodia) were measured according to the manufacturer's instructions. For histology, mouse tissues were fixed in 4% paraformaldehyde and paraffin embedded. Sections (8 μm) were used for haematoxylin and eosin staining.

Glycosphingolipid (GSL) extraction and thin-layer chromatography (TLC)

Extraction and identification of GSLs were performed as described previously with modification (Seyrantepe et al., 2010). Briefly, tissue homogenate or lysosomes was extracted in chloroform:methanol 1:2 (v/v). After centrifugation at 1,000 g for 15 min, the upper phase containing GSLs was isolated and passed through a Bond Elut C18 column (Agilent). Finally, GSLs were eluted using methanol and evaporated by a Centrifugal Vacuum Concentrator. The residue was resuspended in 0.1 ml of chloroform/methanol (1:2, v/v) and further analyzed by TLC and mass spectrometry. The purified GSLs were developed on TLC plates of silica gel with chloroform/methanol/0.25% $CaCl_2$ (50:40:10, v/v/v). The GSL spots were visualized by Iodine and identified by scraping, dissolving and further LC-MS determination.

Identification of glycosphingolipids

Identification of the GSL content of whole cell lysates, lysosome fractions and TLC plates was carried out using an UPLC system and Q-Exactive HFX orbitrap mass spectrometer (Thermo Fisher, CA) equipped with a heated electrospray ionization (HESI) probe. Lipid extracts were separated by a CORTECS C18 100 × 2.1 mm 1.9 µm column (Waters). A binary solvent system was used, in which mobile phase A consisted of ACN:H_2O (60:40), 10 mM ammonium acetate, and mobile phase B consisted of IPA:ACN (90:10), 10 mM ammonium acetate. A 25-minute gradient with a flow rate of 250 µl min^{-1} was used. The column chamber and sample tray were held at 45 °C and 10 °C, respectively. Data with mass ranges of m/z 1000-2000 were acquired in negative ion mode with data dependent MS/MS acquisition. The full scan and fragment spectra were collected with resolutions of 70,000 and 17,500 respectively. The source parameters were as follows: spray voltage: 3000 v; capillary temperature: 320 °C; heater temperature: 300 °C; sheath gas flow rate: 35 Arb; auxiliary gas flow rate: 10 Arb. Data analysis and lipid identification were performed by the software TraceFinder (Thermo Fisher, CA).

Cell culture

HEK293T, Cos7 and HIEC-6 (ATCC) cells were maintained at 37 ºC and 5% CO_2 in DMEM, containing 10% FBS (HyClone) and 100 mg ml^{-1} penicillin-streptomycin. All cell lines were routinely tested for mycoplasma using a PCR detection kit (Sigma, MP0035).

Isolation of lysosomes from cells and tissues

Lysosomal isolation (LYSISO1, Sigma-Aldrich) was performed according to the manufacturer's instructions. Cells or tissues were homogenized in 1× Extraction buffer and the lysate was centrifuged at 1,000 g for 10 min at 4 °C. The supernatant was then centrifuged at 20,000 g for 20 min at 4 °C. The pellet was the crude lysosomal fraction (CLF). The CLF was diluted to the recommended density to build a gradient from 27% Optiprep Density Gradient Medium Solution at the bottom to 8% Optiprep Density Gradient Medium Solution at the top of the tube. The sample was centrifuged at 150,000 g for 4 hr to obtain lysosomes.

Electron microscopy

Isolated lysosomes (LYSISO1, Sigma) were soaked for 24 hours in 4% PFA plus 1% glutaraldehyde in PBS. Then the precipitated pellets were post-fixed with 2% osmium

tetroxide, dehydrated and embedded in Araldite. The negatively stained sample was finally processed for the visualization.

Lysosomal activity assay

Mouse intestinal villi were incubated with Self-Quenched Substrate (ab234622, Abcam) for 2 hrs. After incubation, villi were collected for analysis by flow cytometer. The lysosomal activity was measured by comparing the histograms from flow cytometric analysis.

Measurement of pH

The small intestine was washed in PBS and then cut into small pieces. After washing twice in PBS, tissues were digested for 30 min with 20 mM EDTA at 4 °C. The supernatant was centrifuged at 300 g for 5 min at 4 °C. The pellet was resuspended in DMEM containing 1 μM Lysosensor probe (L7535, Thermo) and incubated for 30 min at 37 °C. After washing once, the pellet was resuspended in PBS for measurement by flow cytometer**.** To measure cellular pH, HEK293T cells expressing the MFSD12 PM mutant (L253A/L254A) were incubated in 10 μM pHrodo Green AM Intracellular pH indicator (P35373, Thermo) for 30 min at 30°C and harvested for analysis after 10 min addition of 5 μM or 10 μM [$^{13}C_3$,^{15}N] cysteine (CNLM-3871-H-PK, Cambridge Isotopes).

Orientation of MFSD12 on lysosomal membranes

Lysosomes were isolated from HEK293T cells with stable expression of FLAG-MFSD12-HA and LAMP1-HA. For labeling of unpermeabilized lysosomes, primary antibodies FLAG (F1804, Sigma), HA (MMS-101P, COVANCE), LAMP1 (ab208943, Abcam) and the corresponding IgG were diluted 1:250 in 1 × Extraction buffer overnight at 4 °C. For labeling of permeabilized lysosomes, the crude lysosomal fraction was fixed in 4% PFA for 30 min at 4 °C. Then the pellet was incubated in 0.1% Triton X-100 in PBS for 10 min at room temperature and incubated with primary antibodies overnight at 4 °C. After washing once in PBS, the lysosomes were further incubated with secondary antibodies for 30 min at 4 °C and then measured by flow cytometry.

Measurement of cysteine and cystine contents

Rapid lysosome immunoprecipitation was performed as previously reported with modification (Adelmann et al., 2020). After 48-hr transfection, HEK293T cells were scraped in KPBS (136 mM KCl, 10 mM KH_2PO_4). The cells were homogenized, then centrifuged at 3,000 g for 2 min at 4 °C. The supernatants were incubated with KPBS-washed magnetic anti-HA beads (88836, ThermoFisher) for 4 min at 4 °C. The pulled down beads were resuspended in 50 μl 80% methanol for measurement by mass spectrometry.

The LC-MS/MS system was a 6500plus QTrap mass spectrometer (AB SCIEX, USA) coupled with an ACQUITY UPLC H-Class system (Waters, USA). Chromatographic separation was achieved using an ACQUITY UPLC BEH Amide column (2.1 × 100 mm, 1.7 μm; Waters). Mobile phase A contained HPLC-grade H_2O-ACN 5/95 (v/v) with 7.5 mM ammonium formate, and mobile phase B was H_2O-ACN 50/50 (v/v) with 7.5 mM ammonium formate. The linear gradient was: 0-1 min, 20% B; 1-3 min, 20%-95% B; 3.1-4 min, 95% B; 4.1-5 min, 20% B. Flow rate was 0.5 mL min^{-1}. Column chamber and sample tray were held at 40 °C and 10 °C, respectively. Data were acquired in multiple reaction monitor (MRM) mode for cysteine, cystine and [$^{13}C_3$,^{15}N] cysteine (CNLM-3871-H-PK, Cambridge Isotopes) with transitions of 121.8/58.9, 241.1/152.0 and 126.0/60.9, respectively. The ion transitions were optimized using chemical standards. The nebulizer

gas (Gas1), heater gas (Gas2), and curtain gas were set at 50, 50, and 35 psi, respectively. The source voltage was 5500 V for positive ion mode. The optimal probe temperature was determined to be 550 °C. SCIEX OS 1.6 software was applied for metabolite identification and peak integration.

Cysteine uptake

The cysteine uptake assay was performed as previously reported with modification (Adelmann et al., 2020). HEK293T cells expressing the plasma membrane (PM)-localized MFSD12 mutant (L253A/L254A) were incubated in uptake buffer (KPBS supplemented with 125 mM sucrose, 2.5 μM Erastin, 4 mM leucine and 3 mM serine) with 2.5 μM [$^{13}C_3$,^{15}N] cysteine for 10 min at 30 °C. The experiment was performed with the pH of the buffer adjusted to 5.6, 6.1, 6.5, 6.9 and 7.4 The cells were collected by centrifugation at 1,000 g for 1 min at 4 °C. After washing twice in cold PBS, 75 μl 80% methanol was added and level of [$^{13}C_3$,^{15}N] cysteine in the cell extract was determined by mass spectrometry.

Plasmids

A mouse *Glb1* promoter fragment spanning from -736 to +40 was cloned upstream of the luciferase gene (pLVX-puro). *Mfsd12* was amplified from a mouse liver tissue cDNA library. A mouse *Lamp1* cDNA clone (MR225631) was purchased from OriGene Technologies, Inc. The shRNA interference construct for human *MFSD12* was in pLKO.1-puro vector. The shRNA sequence was: CCCATTCTCAACTCTAATCCA. All expressed constructs used in this study were confirmed by sequencing.

siRNA screening

To identify which lysosomal transporter affects *Glb1*-Luc, HIEC-6 cells with stable expression of *Glb1*-Luc were transfected with RSV-Luc and siRNAs for human lysosomal transporter-encoding genes selected from ON-TARGET plus genome-wide siRNA libraries (Dharmacon). 48 hr after addition of siRNA, luciferase assays were performed using the Dual Luciferase Reporter Assay System (E1980, Promega) and normalized to co-transfected RSV-Luc activity.

Immunoblotting and immunostaining

Assays were performed as previously described (Han et al., 2015; Chen et al., 2017; Li et al., 2019). For immunoblotting, cells or mouse tissues were homogenized in cell lysis buffer (150 mM NaCl, 50 mM HEPES pH 7.4, 1% Triton X-100). Protein concentrations were determined using the BCA Protein Assay Kit (23227, Thermo Fisher). Samples were loaded on SDS-PAGE gels and then transferred to nitrocellulose membranes. Immunoblotting was done in gelatin buffer (50 mM Tris HCl pH 7.4, 150 mM NaCl, 5 mM EDTA, 0.05% Tween-20) with the corresponding antibodies. Antibodies were purchased and diluted as follows: anti-TFEB (A303-673A, 1:1000), Bethyl; anti-pS6K (9205S, 1:1000), anti-S6K (2708S, 1:1000), anti-LC3 (83506T, 1:1000), anti-P62 (88588T, 1:1000) and anti-HSP90 (4874, 1:1000), Cell Signaling Technology. The rat monoclonal anti-MFSD12 (1:1000) antibody was generated and purified by Abclonal.

For immunostaining assays, cells or paraffin-embedded tissue slices (5 μm) were fixed in 4% paraformaldehyde (PFA) solution and blocked in PBS with 0.2% Triton X-100 and 5% BSA. Samples were stained overnight with specific primary antibodies in blocking solution at 4 ºC, washed three times in PBS and then incubated at room temperature with fluorescent dyes in blocking solution for 1 hr. Antibodies were diluted as follows: anti-LAMP1 (A2582, 1:200), anti-GFP (AE012, 1:200), and anti-ALPI (A6226, 1:250), Abclonal; anti-HA (MMS-101P, 1:1000), COVANCE. Samples were incubated in DAPI solution for

10 min to stain the DNA, and then coverslipped. Tiled images were obtained using an epifluorescence microscope (Zeiss) and the exposure time for each channel was kept constant for all slides.

Quantitative PCR

Total RNA from cells or mouse tissues was extracted using a Total RNA Purification kit (Omega). cDNA was obtained using the RevertAid First Strand cDNA Synthesis kit (Thermo). RNA levels were measured with the LightCycler 480 II (Roche) as previously described (Han et al., 2015; Chen et al., 2017; Li et al., 2019). The following primers were used for qPCR:

Human *ACTB*-forward: 5’- TGCTATCCCTGTACGCCTCT-3’

Human *ACTB*-reverse: 5’- CTCCTTAATGTCACGCACGA-3’

Human *MFSD12*-forward: 5’- AGGCTCATCGTGAACCTGTC-3’

Human *MFSD12*-reverse: 5’-GACAAGAAGCCGCTGAGGTA-3’

Mouse *Mfsd12*-forward: 5’-GGGCTGCCTTGCTGTACTAT-3’

Mouse *Mfsd12*-reverse: 5’-CACCTTCTCGTGGTCACTGG-3’

Mouse *Rpl32*-forward: 5’-TCTGGTGAAGCCCAAGATCG-3’

Mouse *Rpl32*-reverse: 5’-CTCTGGGTTTCCGCCAGTT-3’

Statistical methods

Age-matched mice were randomly assigned for the experiments. The animal numbers used for all experiments are outlined in the corresponding figure legends. All studies were performed on at least three independent occasions. Results are reported as mean ± SEM. Comparison of different groups was carried out using the two-tailed unpaired Student’s *t*-test or two-way ANOVA. Differences were considered statistically significant at $P < 0.05$.

References

Adelmann, C.H., Traunbauer, A.K., Chen, B., Condon, K.J., Chan, S.H., Kunchok, T., Lewis, C.A., and Sabatini, D.M. (2020). MFSD12 mediates the import of cysteine into melanosomes and lysosomes. Nature 588, 699-704.

Chen, L., Wang, K., Long, A., Jia, L., Zhang, Y., Deng, H., Li, Y., Han, J., and Wang, Y. (2017). Fasting-induced hormonal regulation of lysosomal function. Cell Res 27, 748-763.

Han, J., Li, E., Chen, L., Zhang, Y., Wei, F., Liu, J., Deng, H., and Wang, Y. (2015). The CREB coactivator CRTC2 controls hepatic lipid metabolism by regulating SREBP1. Nature 524, 243-246.

Li, E., Shan, H., Chen, L., Long, A., Zhang, Y., Liu, Y., Jia, L., Wei, F., Han, J., Li, T.*, et al.* (2019). OLFR734 Mediates Glucose Metabolism as a Receptor of Asprosin. Cell Metab 30, 319-328 e318.

Seyrantepe, V., Lema, P., Caqueret, A., Dridi, L., Bel Hadj, S., Carpentier, S., Boucher, F., Levade, T., Carmant, L., Gravel, R.A.*, et al.* (2010). Mice doubly-deficient in lysosomal hexosaminidase A and neuraminidase 4 show epileptic crises and rapid neuronal loss. PLoS Genet 6, e1001118.

Figure S1

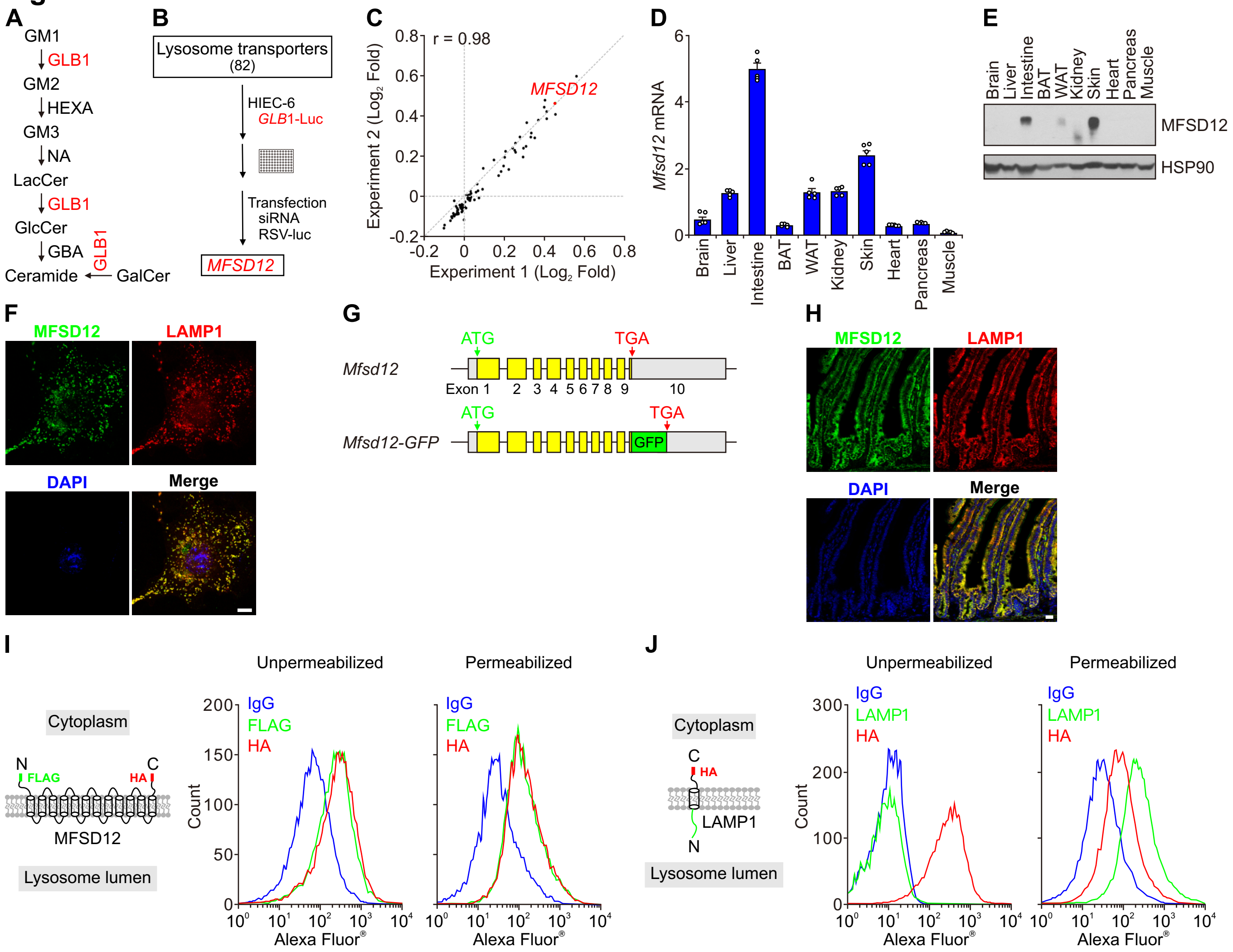

Figure S2

A

Mouse	Gender	Genotype	Number	Ratio (%)
E14.5	Male	WT	37	53
		CKO	33	47
	Female	WT	36	52
		CKO	33	48
E18.5	Male	WT	39	80
		CKO	10	20
	Female	WT	42	76
		CKO	13	24
P1	Male	WT	70	84
		CKO	13	16
	Female	WT	78	79
		CKO	21	21
P21	Male	WT	123	88
		CKO	17	12
	Female	WT	125	82
		CKO	27	18
P56	Male	WT	123	90
		CKO	13	10
	Female	WT	125	84
		CKO	24	16

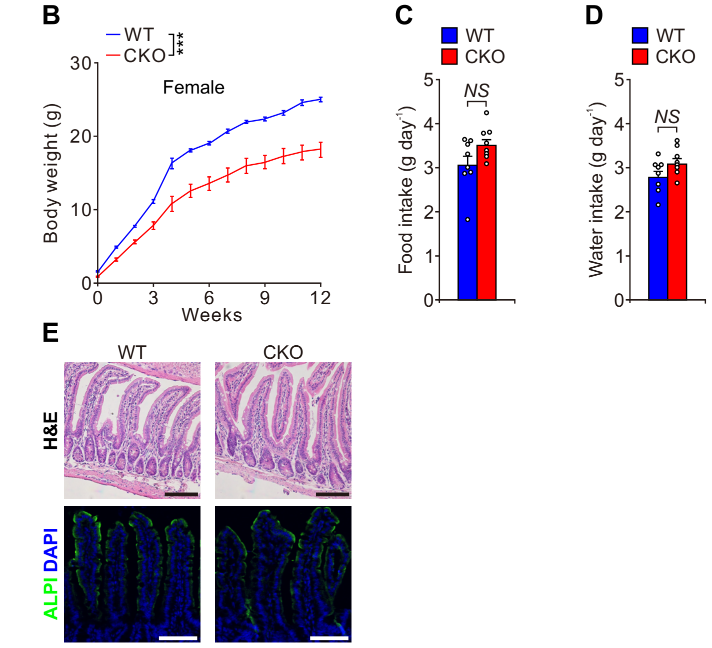

Figure S3

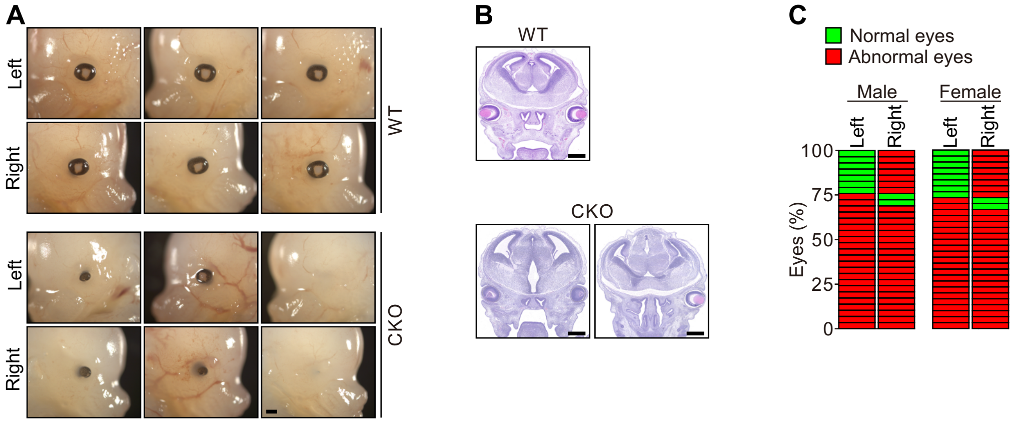

Figure S4

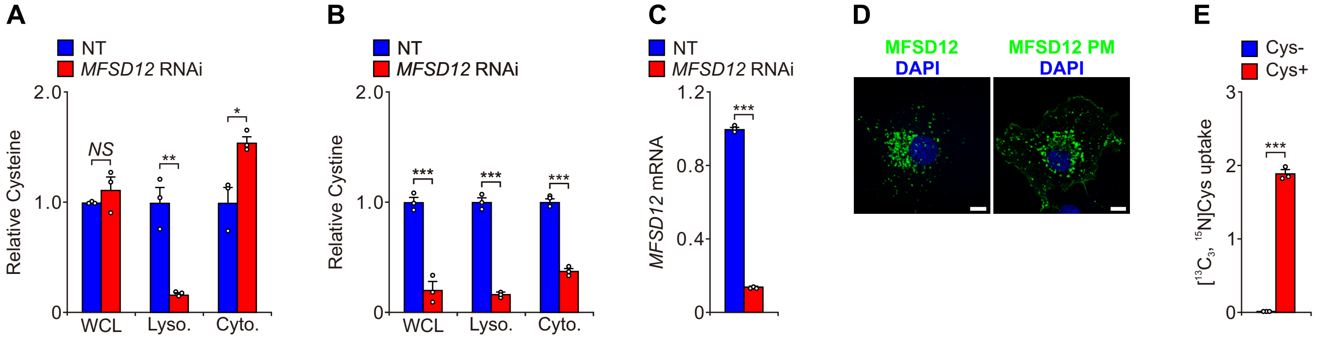

Supplement: pwac034_suppl_Supplementary_Material [file pwac034_suppl_supplementary_material.pdf]
